# A facility for laboratory mice with a natural microbiome at Charité – Universitätsmedizin Berlin

**DOI:** 10.1038/s41684-024-01474-4

**Published:** 2024-11-12

**Authors:** Natascha Drude, Stefan Nagel-Riedasch, Stephan P. Rosshart, Andreas Diefenbach, The “Charité 3R Wildling Mouse Model in Health and Disease (C3R Wildling HeaD)” consortium, Natascha Drude, Andreas Diefenbach, Kai Diederich, Claudia U. Duerr, Christoph Harms, Frank Heppner, Marina Kolesnichenko, Lars Lewejohann, Marcus A. Mall, Dominik Müller, Bastian Opitz, Stephan P. Rosshart, Gilbert Schönfelder, Ulf Tölch, Gerald Willimsky, Stefan Jordan, Stefan Jordan

**Affiliations:** 1 Berlin Institute of Health (BIH) at Charité – Universitätsmedizin Berlin, BIH QUEST Center for Responsible Research, Berlin, Germany; 2 Charité – Universitätsmedizin Berlin, corporate member of Freie Universität Berlin and Humboldt-Universität zu Berlin, Forschungseinrichtungen für Experimentelle Medizin/FEM, Berlin, Germany; 3 Department of Microbiome Research, University Hospital Erlangen, Friedrich-Alexander-Universität Erlangen-Nürnberg (FAU), Erlangen, Germany; 4 Department of Medicine II, Medical Center - University of Freiburg, Faculty of Medicine, Freiburg, Germany; 5 Charité – Universitätsmedizin Berlin, corporate member of Freie Universität Berlin and Humboldt-Universität zu Berlin, Institute of Microbiology, Infectious Diseases and Immunology, Berlin, Germany; 6 Department of Infectious Diseases, Medical Microbiology and Hygiene, Heidelberg University, Heidelberg, Germany; 7 German Federal Institute for Risk Assessment (BfR), German Centre for the Protection of Laboratory Animals (Bf3R), Max-Dohrn-Str. 8-10, 10589 Berlin, Germany; 8 Charité – Universitätsmedizin Berlin, corporate member of Freie Universität Berlin and Humboldt-Universität zu Berlin, Klinik und Hochschulambulanz für Neurologie, Department of Experimental Neurology, Berlin, Germany; 9 Charité – Universitätsmedizin Berlin, corporate member of Freie Universität Berlin and Humboldt-Universität zu Berlin, Center for Stroke Research Berlin, Berlin, Germany; 10 Einstein Center for Neuroscience, Berlin, Germany; 11 German Center for Cardiovascular Research (DZHK), partner site Berlin, Berlin, Germany; 12 Charité – Universitätsmedizin Berlin, corporate member of Freie Universität Berlin and Humboldt-Universität zu Berlin, Department of Neuropathology, Berlin, Germany; 13 Cluster of Excellence, NeuroCure, Berlin, Germany; 14 German Center for Neurodegenerative Diseases (DZNE) Berlin, Berlin, Germany; 15 Charité – Universitätsmedizin Berlin, corporate member of Freie Universität Berlin and Humboldt-Universität zu Berlin, Department of Hepatology and Gastroenterology, CCM CVK, Berlin, Germany; 16 Freie Universität Berlin, Institute of Animal Welfare, Animal Behavior and Laboratory Animal Science, Berlin, Germany; 17 Charité - Universitätsmedizin Berlin, corporate member of Freie Universität Berlin and Humboldt-Universität zu Berlin, Department of Pediatric Respiratory Medicine, Immunology and Critical Care Medicine, Berlin, Berlin, Germany; 18 German Center for Lung Research (DZL), associated partner site, Berlin, Germany; 19 Experimental and Clinical Research Center (ECRC) & Max-Delbrück-Centrum für Molekulare Medizin in der Helmholtz-Gemeinschaft (MDC), Berlin, Germany; 20 Charité – Universitätsmedizin Berlin, corporate member of Freie Universität Berlin and Humboldt-Universität zu Berlin, Department of Infectious Diseases, Respiratory Medicine and Critical Care, Berlin, Germany; 21 Charité - Universitätsmedizin Berlin, corporate member of Freie Universität Berlin, Humboldt-Universität zu Berlin, and Berlin Institute of Health, Institute of Clinical Pharmacology and Toxicology, Division Toxicology, Berlin, Germany; 22 Charité-Universitätsmedizin Berlin, corporate member of Freie Universität Berlin and Humboldt-Universität zu Berlin, Institute of Immunology, Berlin, Germany; 23 German Cancer Research Center, Heidelberg, Germany; 24 German Cancer Consortium, partner site Berlin, Berlin, Germany

**Keywords:** Microbial communities, Immunology

## Abstract

Mice with a natural microbiome are a promising research model for basic and applied science because of their closer resemblance to the human superorganism compared to mice born and raised under stringent hygiene conditions. Consequently, biomedical therapies developed and tested in “Wildling mice” hold great potential for successful translation into clinical applications. Over the past four years, scientists, veterinarians and institutional officials at Charité – Universitätsmedizin Berlin, supported by the University Hospital Erlangen, have designed a facility for Wildling mice and developed a conceptual framework for safe and ethical preclinical research involving mice with a natural microbiome.

Our microbiome, i.e. the multitude of microscopic organisms that colonize humans, is an important factor in the maintenance of health and the development of diseases^1^. However, the standardization of hygiene in animal husbandry, aimed at reducing biological noise and minimizing variability in experimental results, has greatly contributed to the exclusion of natural microbiota from preclinical research environments^2^. Thus, the environmental conditions under which hygienically standardized, specified pathogen-free (SPF) laboratory mice are born and kept differ substantially from the real-world conditions to which humans and animals are normally exposed. These deviating environmental conditions result in physiological discrepancies between humans and laboratory mice that contributed to the failure of some drug candidates, which showed promising results in preclinical research but eventually failed in clinical trials^3,4^.

In 2016, Beura and colleagues demonstrated that SPF mice harboring only a reduced microbiome exhibit a neonatal-like immune status. In contrast, animals exposed to environmental microorganisms, such as mice caught in the wild or purchased from a pet shop, develop a mature immune phenotype comparable to adult humans^5^. This study suggested that mice with a natural microbiome might be a better model for humans in preclinical and translational research compared to laboratory mice born and raised under stringent SPF hygiene conditions. Yet, basic research relies on the well-characterized genetic background of laboratory mouse strains as well as on the multitude of genetic models and tools that exist. Consequently, various strategies have been explored to provide standard laboratory mouse strains with a natural microbiome, including co-housing with feral and pet shop mice^5^, sequential exposure to commensals^6^, keeping the animals in outdoor enclosures^7^ or on bedding from large animals^8^ and fecal transplants from wild mice^9^. Lately, Rosshart and colleagues transferred embryos from laboratory mouse strains into wild-caught mice. During delivery, the laboratory mice would receive the natural microbiome of their surrogate mothers, closely resembling human inoculation during birth^10^. After primary colonization from feral mice, these so-called “Wildling mice” can be bred like any other laboratory mouse strain and the natural microbiome is maintained^10^. Importantly, in two preclinical trials, where rodent and even nonhuman primate models had failed to predict the human response to harmful drug treatments^3,4^, Wildling mice accurately phenocopied human immune responses, suggesting they could have prevented these catastrophically failed human trials at the preclinical stage^10^. Hence, since these laboratory mice with a natural microbiome better resemble the human superorganism, results obtained from experiments with Wildling mice might have a greater potential for both basic and applied research as well as for translation into clinical applications compared to results obtained with SPF mice.

## A collaborative effort to evaluate Wilding mice as a model for preclinical research

To systematically evaluate the usefulness of Wildling mice for preclinical research in comparison to mice bred and kept under standardized hygiene conditions, scientists, veterinarians and institutional officials at Charité – Universitätsmedizin Berlin, the Department of Microbiome Research at the Friedrich-Alexander-Universität Erlangen-Nürnberg (FAU) and the German Centre for Protection of Laboratory Animals (Bf3R) have founded the “Charité 3^R^ Wildling mice in Health and Disease (C3^R^ Wildling HeaD)” consortium. The aim for the next two years is to thoroughly compare Wildling mice and SPF mice in a range of widely used preclinical models for relevant communicable and non-communicable diseases including influenza infection, nosocomial lung infection, acute kidney injury, cystic fibrosis, Alzheimer disease, cancer and stroke. Due to the putative impact of the natural microbiome on the immune system of Wilding mice and subsequently disease outcome, we will perform comprehensive immunophenotyping across the selected diseases. Furthermore, we will test the differential outcome of immunomodulatory therapies in SPF and Wildling mice. Our goal is to determine the external validity of the Wildling mouse model, i.e., the extent to which results obtained in Wildling mice can be generalized and applied to a broader context.

Importantly, the “C3^R^ Wildling HeaD” consortium does not only bundle the research interests of the participating scientists, but also serves as a focus point for other institutional stakeholders with a strong interest in the Wilding mouse model. For example, the consortium is financially supported by the “Charité 3^R^” office (C3^R^), an infrastructure that actively strengthens the implementation of the 3Rs principle (Replacement, Reduction, Refinement) for ethical use of animals in biomedical research^11^. Based on the hypothesis that preclinical research with mice with a natural microbiome and a mature immune system might be more predictive for immune-mediated disease outcome in humans, Wildling mice could improve the evidence generated per animal tested within an experiment, thereby reducing the number of animals used for research.

In the contemporary 3Rs definition of the British national 3Rs centre, ‘Reduction’ refers to “appropriately designed and analysed animal experiments that are robust and reproducible, and truly add to the knowledge base”(see NC3R website). Therefore, to enhance the robustness and reproducibility of preclinical studies conducted in the framework of the “C3^R^ Wildling HeaD” consortium, the participating research groups receive comprehensive support by the Responsible PrecliniX of the Berlin Institute of Health QUEST Center for Responsible Research. Responsible Preclinix is an institutional initiative to assist in robust experimental design including sample size calculations, blinding and randomization. Furthermore, within the “C3^R^ Wildling HeaD” consortium, Responsible PrecliniX is conducting a meta-research study that will compare the Wildling mouse model across all projects to the current gold standard model, the SPF mouse. Accordingly, Responsible PrecliniX will support harmonization of tissue sampling protocols and animal scoring to ensure the consistency and comparability of results. Because pre-registration of animal experiments could substantially improve the transparency and accountability of biomedical studies and animal welfare^12^, all studies conducted in the framework of the “C3^R^ Wildling HeaD” consortium need to be pre-registered according to the common standards for the pre-registration in animal research^13^.

The support of research using Wildling mice by the responsible office for animal husbandry and experimental medicine at Charité – Universitätsmedizin Berlin, “Forschungseinrichtungen Experimentelle Medizin (FEM)”, was a prerequisite for the entire project. From the experimental animal science point of view, the microbiome is a complex influential variable in animal experimentation. Though it is known that the complexity of the microbiome differs significantly between SPF facilities^14,15^, strategies for the control and standardization of the microbiome between scientific institutions, analogous to the harmonization of the genetic background or other husbandry parameters, are virtually non-existent for now. In contrast to reductionist approaches such as gnotobiotes, the Wildling mice model combines a uniform genetic background with a complex, real-life microbiome. In this context, the variance in the natural microbiome could be regarded as systematic heterogenization. Thus, research with Wildling mice could increase the robustness and generalizability of preclinical results and contribute to a better reproducibility of animal research^16,17^.

However, the natural microbiome of Wildling mice includes bacteria, viruses and parasites such as highly transmissible worms that pose a challenge to husbandry facilities maintaining an overall SPF hygiene level. Hence, we needed to create a structure for experimentation with Wildling mice that includes stringent biocontainment measures to protect areas of the animal facility where SPF mice are bred and maintained.

## Design of a facility for safe and ethical research with Wildling mice

**Fig. 1:**
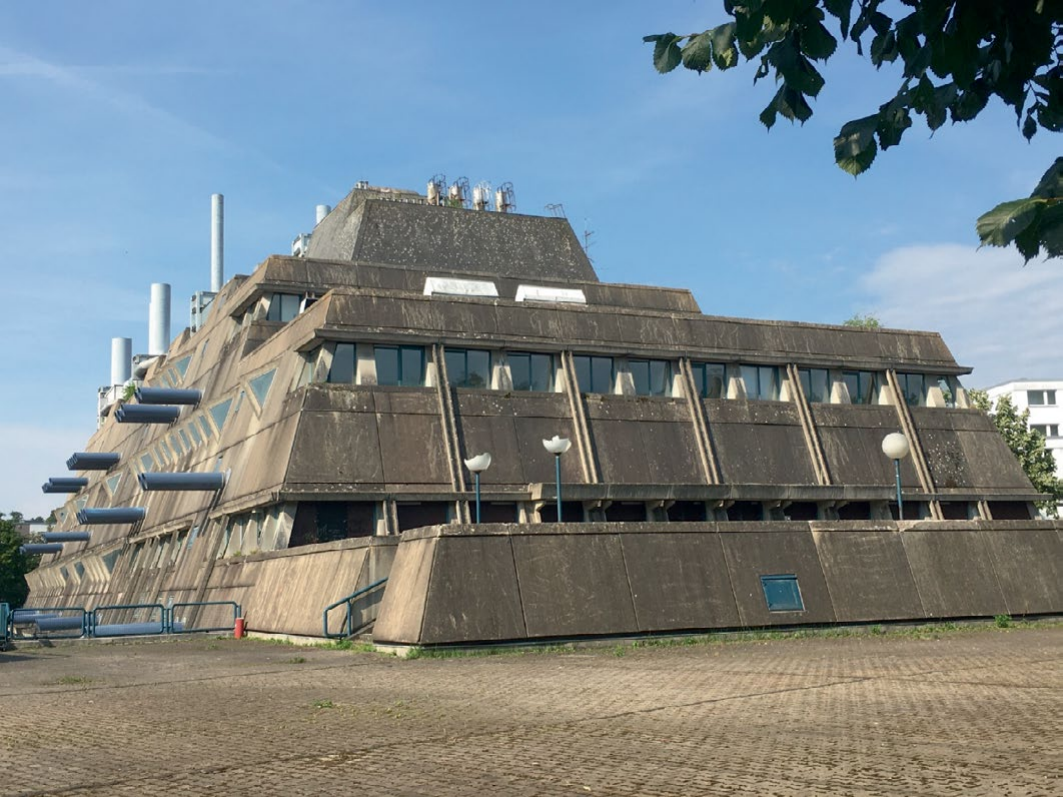
The “Mäusebunker” at Charité – Universitätsmedizin Berlin. First experiments with Wildling mice took place in the “Mäusebunker” (“mouse bunker”) shortly before it was shut down in 2020. Today, the building is a listed monument as an outstanding example of brutalist architecture. Stefan Jordan

**Fig. 2:**
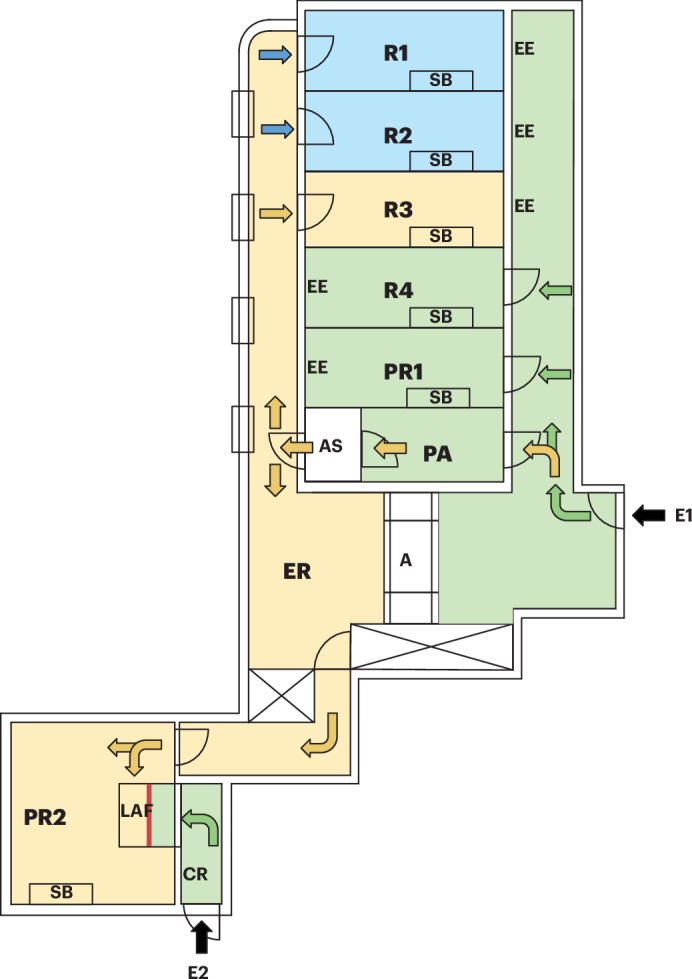
Design of the facility for Wildling mice. Black arrows indicate facility entrances. E1: Facility entrance. E2: access to the laminar airflow cabinet from outside of the facility. Green areas: SPF hygiene standards. Green arrows represent routes accessible when working with SPF animals. Yellow areas: Wildling mice. Yellow arrows indicate paths available for working with Wildlings after air showering. Blue areas: Wilding mice breeding rooms. Blue arrows indicate access for husbandry staff only. PA: personnel air lock with air shower. AS: air shower. R1, R2: rooms for breeding of Wildling mice. R3: room for keeping of Wilding mice. R4: room for keeping of SPF mice. PR1: procedure room for SPF mice. PR2: procedure room for Wildling mice. SB: sterile working bench. EE: emergency exits. A: autoclave. ER: equipment room. LAF: laminar airflow cabinet for interventions under protective airflow. CR: changing room in front of LAF cabinet. The red line marks the glass wall inside the LAF cabin, which divides the space into two sections, which can either be accessed from E1 via PR2 or from E2 via CR. Adapted from Drude et al. *JoVE*^19^.

The conceptualization of a facility for Wildling mice at Charité – Universitätsmedizin Berlin started four years before it was eventually operational and the project was supported by concepts developed by Rosshart and colleagues at the Department of Microbiome Research, University Hospital Erlangen, FAU. First experiments with Wildling mice were performed in the iconic “Mäusebunker” (“mice bunker”), the primary animal husbandry facility of the FEM, shortly before it was shut down in 2020 (Fig. 1). These initial experiments required the development of biocontainment measures and provided first-hand experiences of experimentation with Wildling mice that were later incorporated into the concept of the facility for Wildling mice. To create the current facility, an existing mouse husbandry was extensively reconstructed (Fig. 2). Though unnecessary for Wilding mice per se, the facility has biosafety level (BSL) 2 to allow users to work with infection models which require this level of biosafety.

Of course, strict adherence to biocontainment measures while working in the facility for Wildling mice is mandatory to prevent the spread of microorganisms to other areas of the animal facility. This includes restricting access to the facility for Wildling mice to as few people as possible per research group. Before receiving access to the facility, a personal introduction given by the institutional animal welfare officer is obligatory. Furthermore, we have created a structured communication platform, the "Wildling Wiki", which serves as a repository for Standard Operating Procedures (SOPs) and other useful information for work inside the facility^18^.

The facility provides procedure rooms that can be booked through an online booking system, ensuring smooth working processes and a limited number of people inside the facility. People who have previously entered the facility for Wildling mice are prohibited from entering any other animal facility on the same day. Therefore, specific animal husbandry staff are assigned exclusively to the facility for Wildling mice, providing standardized and contemporary housing conditions, e.g., using a tunnel or a cup to handle the animals to reduce distress.

Because some experimental setups involve both Wildling mice and SPF mice, we wanted to keep mice of both hygiene states in close proximity to ensure similar experimental conditions. Therefore, the facility contains two areas that are separated by an air shower (Fig. 2). Both areas have designated rooms for breeding, housing and experimental procedures. Between the rooms of the respective areas, mice are transferred in closed individually ventilated cages (IVC). The cages can only be opened under class II work benches to ensure microbial containment and work safety. If the experiment includes both Wildling mice and SPF mice, mice in the SPF area must be handled first. The Wildling mice area is operated as a negative pressure higher safety level housing barrier with all the necessary personal hygiene and sanitation protocols normally foreseen in such areas.

The founder pairs of Charité’s Wildling mice colony were imported from the Wilding mice colony established at the Department of Microbiome Research, University Hospital Erlangen, FAU, and were tested for an extended panel of microbial agents to exclude zoonotic pathogens exceeding BSL 2 level. Breeding of mice is performed in standard IVC in pairs. Weaned Wildling mice offspring are pooled in IVC in groups of 20–25 mice to support microbial equilibration. These cages receive worm humus soil and hay commercially available from the pet shop for microbial enrichment. Afterwards, mice are kept in in groups with a maximum of five mice. Depending on the experimental requirements, mice can as well be housed in different cage systems. Though unnecessary for Wildling mice, they are provided with steam-sterilized bedding and enrichment items facilitating the work routine in the facility.

**Fig. 3:**
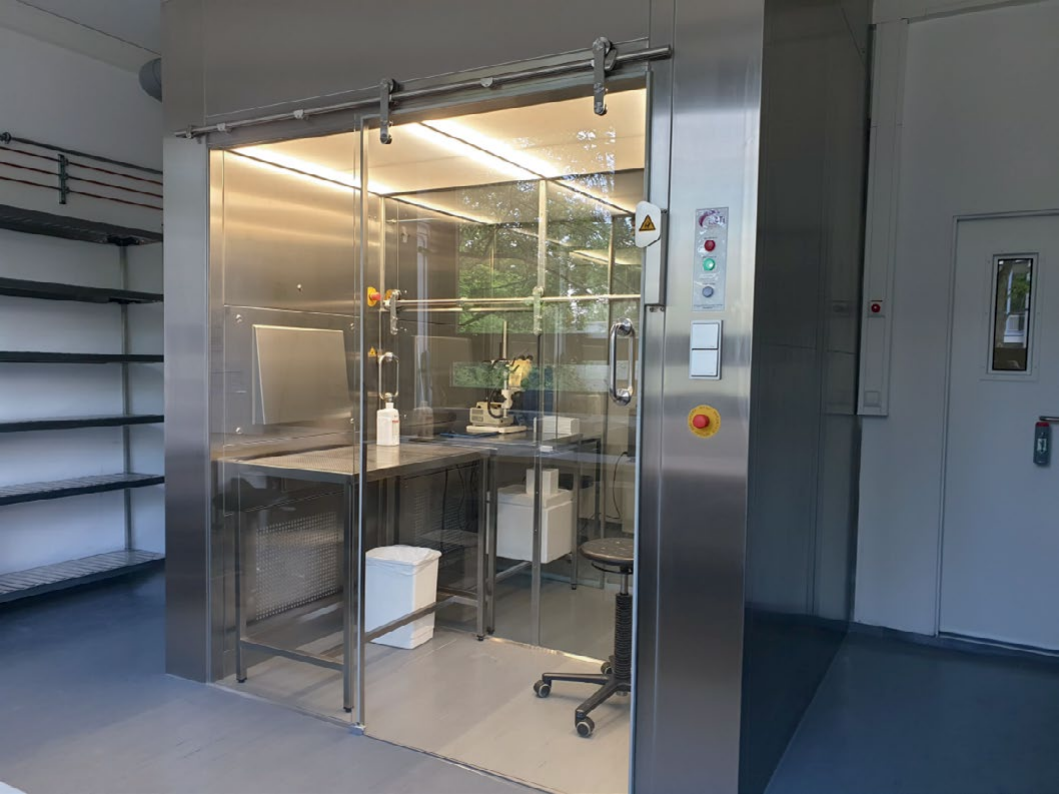
The Laminar Air Flow (LAF) cabinet. The LAF cabinet serves as a sterile intervention room for procedures involving Wildling mice and as an air lock to export samples from the Wildling area. Anke Lohan

The main procedure room for Wildling mice is equipped with a two-compartment Laminar Air Flow (LAF) cabinet which serves as a sterile intervention room for the dissection and surgery of Wildling mice and as an air lock to export samples from the Wildling mice area (Fig. 3). Furthermore, the LAF cabinet serves as transfer bench for embryonic transfer that will be used to generate genetically modified Wildling mouse lines corresponding to existing SPF mouse lines.

After exporting samples from the Wildling mice facility, further processing can be performed in adjacent laboratories. Spatial proximity enables researchers to process time-sensitive materials on-site and limits the institutional locations where samples from Wildling mice are handled.

It is important to note that the more complex Wildling model may not be equally relevant for all research questions or experimental disease models. Nevertheless, the use of Wildling mice in biomedical research could improve the quality of science as the results become more generalizable and reproducible. Because Wildling mice seem to better reflect human immune traits, promising preclinical results obtained in Wildling mice hold greater potential for clinical translation, potentially expediting drug development. At the same time, the use of more appropriate animal models based on Wildling mice will have a positive impact on animal welfare, as the preference for more relevant mouse models will generally lead to a reduction in unnecessary animal testing worldwide. Therefore, we aim to facilitate research with Wildling mice at other research institutions. We welcome requests from scientists and institutions for scientific collaborations or support in the implementation of research with Wilding mice.

## References

[1] Lynch, S. V. & Pedersen, O. *N. Engl. J. Med.* **375**, 2369–2379 (2016). 27974040 10.1056/NEJMra1600266

[2] Voelkl, B., Wurbel, H., Krzywinski, M. & Altman, N. *Nat. Methods* **18**, 5–7 (2021). 33408399 10.1038/s41592-020-01036-9

[3] Fisher, C. J. Jr. et al. *N. Engl. J. Med.* **334**, 1697–1702 (1996). 8637514 10.1056/NEJM199606273342603

[4] Suntharalingam, G. et al. *N. Engl. J. Med.* **355**, 1018–1028 (2006). 16908486 10.1056/NEJMoa063842

[5] Beura, L. K. et al. *Nature* **532**, 512–516 (2016). 27096360 10.1038/nature17655 PMC4871315

[6] Reese, T. A. et al. *Cell Host Microbe* **19**, 713–719 (2016). 27107939 10.1016/j.chom.2016.04.003 PMC4896745

[7] Leung, J. M. et al. *PLoS Biol.* **16**, e2004108 (2018). 29518091 10.1371/journal.pbio.2004108 PMC5843147

[8] Sbierski-Kind, J. et al. *Front. Immunol.* **9**, 1069 (2018). 29892281 10.3389/fimmu.2018.01069 PMC5985496

[9] Rosshart, S. P. et al. *Cell* **171**, 1015–1028 e1013 (2017). 29056339 10.1016/j.cell.2017.09.016 PMC6887100

[10] Rosshart, S. P. et al. *Science* **365**, eaaw4361 (2019). 31371577 10.1126/science.aaw4361 PMC7377314

[11] Burch, R.L. & Russell, W.M.S. The Principles of Humane Experimental Technique. (Methuen & Co. Limited, London; 1959).

[12] Heinl, C. et al. *EMBO Rep.* **21**, e49709 (2020). 31867805 10.15252/embr.201949709 PMC6945056

[13] Heinl, C. et al. *PNAS Nexus* **1**, pgac016 (2022). 36712788 10.1093/pnasnexus/pgac016 PMC9802105

[14] Yoon, Y. M. et al. *Respir. Res.* **23**, 337 (2022). 36496380 10.1186/s12931-022-02264-7 PMC9741526

[15] Yamamoto, M. L. et al. *Cancer Res.* **73**, 4222–4232 (2013). 23860718 10.1158/0008-5472.CAN-13-0022 PMC3718495

[16] Voelkl, B. et al. *Nat. Rev. Neurosci.* **21**, 384–393 (2020). 32488205 10.1038/s41583-020-0313-3

[17] Carneiro, C. F. D., Drude, N., Hülsemann, M., Collazo, A. & Toelch, U. *Expert Opin. Drug Discov.* **18**, 1273–1285 (2023). 37691294 10.1080/17460441.2023.2251886

[18] Dirnagl, U., Kurreck, C., Castanos-Vélez, E. & Bernard, R. *EMBO Rep.* **19**, e47143 (2018). 30341068 10.15252/embr.201847143 PMC6216282

[19] Drude, N. et al. *JoVE* In Press.

